# Identification of a Polymorphism in the N Gene of SARS-CoV-2 That Adversely Impacts Detection by Reverse Transcription-PCR

**DOI:** 10.1128/JCM.02369-20

**Published:** 2020-12-17

**Authors:** Manu Vanaerschot, Sabrina A. Mann, James T. Webber, Jack Kamm, Sidney M. Bell, John Bell, Si Noon Hong, Minh Phuong Nguyen, Lienna Y. Chan, Karan D. Bhatt, Michelle Tan, Angela M. Detweiler, Alex Espinosa, Wesley Wu, Joshua Batson, David Dynerman, Debra A. Wadford, Andreas S. Puschnik, Norma Neff, Vida Ahyong, Steve Miller, Patrick Ayscue, Cristina M. Tato, Simon Paul, Amy L. Kistler, Joseph L. DeRisi, Emily D. Crawford

**Affiliations:** a Chan Zuckerberg Biohub, San Francisco, California, USA; b University of California, San Francisco, California, USA; c Chan Zuckerberg Initiative, Redwood City, California, USA; d California Department of Public Health, Richmond, California, USA; e Department of Public Health, Madera, California, USA; Rhode Island Hospital

**Keywords:** COVID-19, PCR, SARS-CoV-2, diagnosis, mutation, polymorphism, sensitivity

## LETTER

Since 7 April 2020, our coronavirus disease 2019 (COVID-19) diagnostic laboratory (CLIAHUB) has received samples from multiple counties in California. Our reverse transcriptase PCR (RT-PCR) protocol (1) employs N-gene (NIID_2019-nCov_N_F2/R2ver3/P2 [Japan]) (2) and E-gene (E_Sarbeco_F/R/P1 [Germany]) (3) simplex assays. In July 2020, we identified 40+ samples from Madera County with poor N-gene assay performance relative to the E-gene assay.

**FIG 1:**
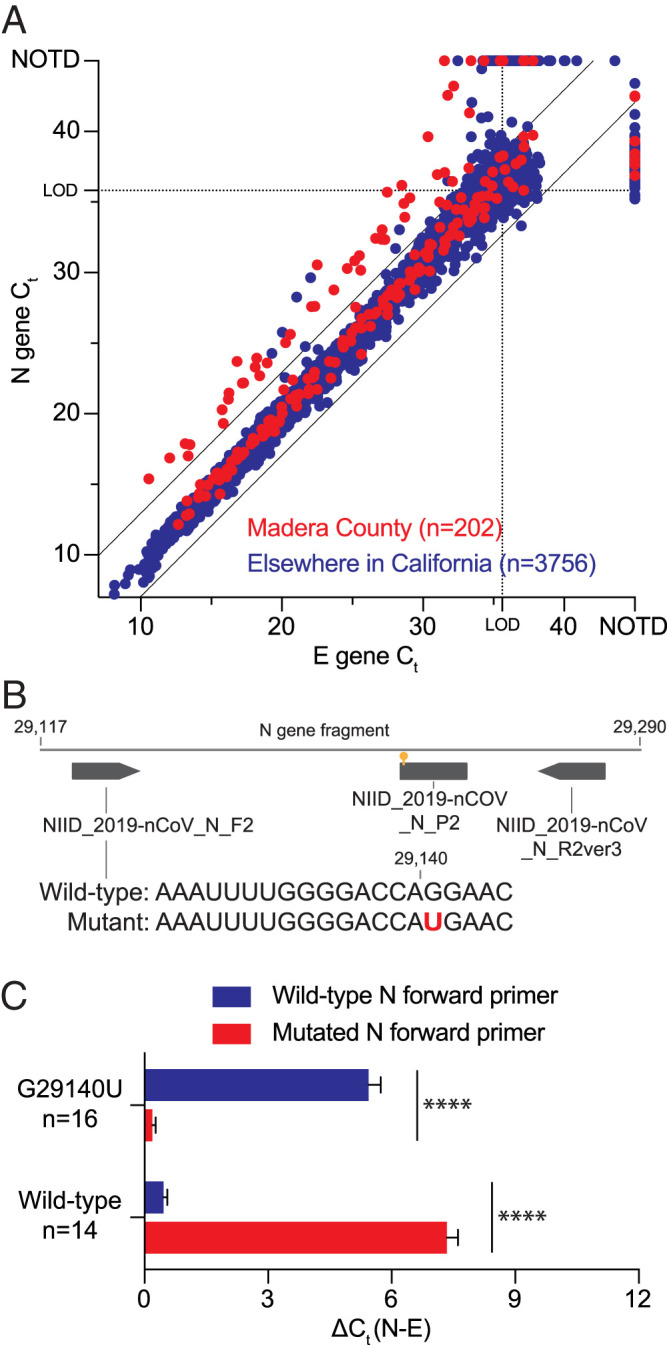

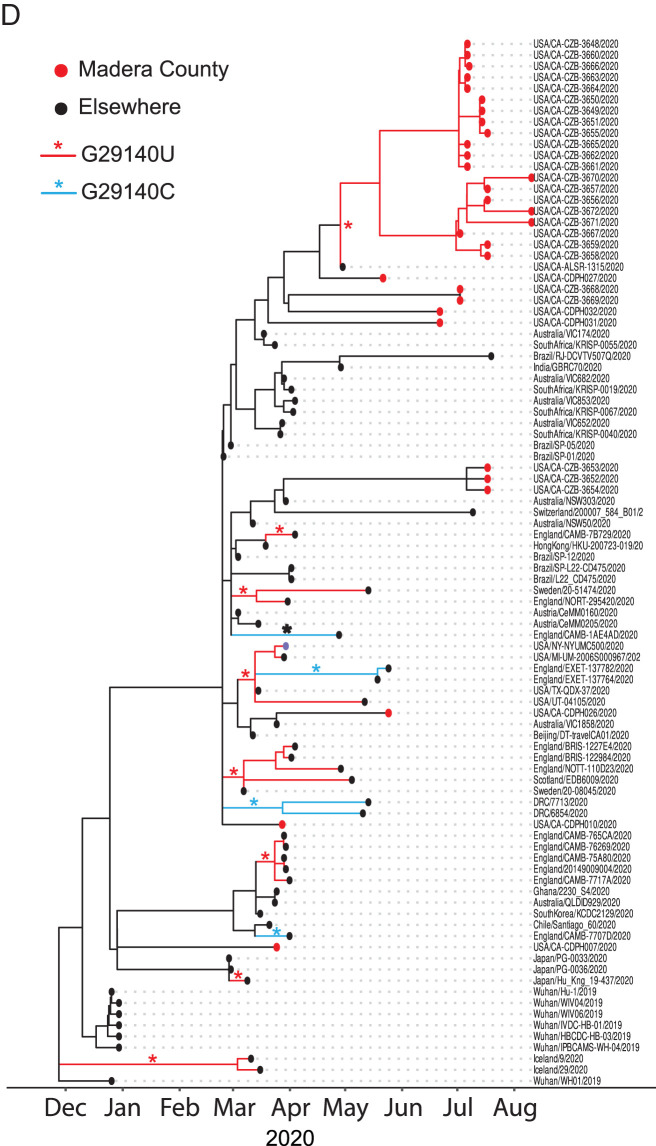
Single mutation in forward N-gene primer binding site, prevalent across the world, decreased SARS-CoV-2 RT-PCR sensitivity. (A) Potential SARS-CoV-2 mutants were identified by their increased Δ*C_T_* between the N- and E-gene assays (>2.5× standard deviation of average Δ*C_T_*; cutoff indicated by black lines). Dotted lines indicate the average *C_T_* value at the limit of detection (LOD) of each assay, above which more variation is expected. NOTD, not detected. (B) Diagram showing a fragment of the N gene, with the N-gene primers and probe originally developed by the National Institute of Infectious Diseases (NIID) in Tokyo, Japan (4), and the identified G29140U mutation indicated. (C) The increased Δ*C_T_*(N−E) of mutant lines using the conventional RT-PCR with the wild-type primer was reversed when a primer incorporating the mutation was used. The opposite was observed for wild-type samples that showed an increased Δ*C_T_*(N−E) when the mutated primer was used, further validating causality of the G29140U mutation for reduced N-gene RT-PCR performance. Error bars indicate standard errors of the means. ****, significant difference determined by a *t* test (*P* < 0.0001). (D) Phylogeny of SARS-CoV-2 isolates with N-gene mutation, including those with the G29140U mutation. Inferred mutation events on the tree are annotated with an asterisk that is colored depending on the allele. Both synonymous variants of the Q289H mutant are found, with the mutation estimated to have recurred 11 times on the tree, and only one of the mutant samples from GISAID was identical by descent to the Madera cluster. One of the wild-type Madera samples was closely related to the mutant cluster, with a common ancestor just before the mutation event. Sequence data are available in Table S1. All code used for analyses and figure generation is described in Text S1.

Figure 1A shows the concordance of cycle threshold (*C_T_*) values for both assays in the 3,958 positive tests conducted during 27 May to 7 August 2020. For samples with positive E- and N-gene results (*n* = 3,629), the N- and E-gene *C_T_* value difference [Δ*C_T_*(N−E)] was 0.40 ± 1.18 (mean ± standard deviation).

Sequencing of the detected N-gene fragments of 57 samples with a Δ*C_T_*(N−E) of ≥2.96 (2.5 standard deviations above the mean) identified 46 samples (45 from Madera) to have a G29140U mutation located in the forward primer binding site (16th of 20 nucleotides) of the N-gene assay (Fig. 1B). In 5 mutant samples, the N gene was undetectable by RT-PCR, but these cases were still recognized as positive for severe acute respiratory syndrome coronavirus 2 (SARS-CoV-2) by the E-gene assay. The 11 wild-type samples with an increased Δ*C_T_*(N−E) are considered rare artifacts.

When the RT-PCR was repeated using a forward primer with full complementarity to the mutant sequence (Fig. 1C), the mean Δ*C_T_*(N−E) of 16 randomly selected mutant samples dropped from 5.44 with the canonical primer to 0.19 with the mutated primer. This trend was inverted for the 14 randomly selected wild-type samples where the Δ*C_T_*(N−E) increased from 0.46 to 7.34 with the canonical and mutated primer, respectively. These data validate causality of G29140U for the observed aberrant *C_T_* values of the N-gene assay, reducing its sensitivity by 67-fold.

G29140U encodes a Q289H amino acid mutation in the N gene that was also found in 27 other sequences available on GISAID (4), showing worldwide occurrence of these mutants. Q289H is located within the dimerization domain of the nucleocapsid protein but is not involved in any known dimer interface interactions, though tertiary-structure-level interactions could be impacted by mutations at this position (5).

Whole-genome sequencing of randomly selected mutant (*n* = 20) and wild-type (*n* = 11) samples from Madera showed little genetic diversity between our mutant samples and revealed that a GISAID sequence from San Diego was identical by descent (Fig. 1D). The remaining 26 mutants from GISAID fell on different clades of the tree, with 11 estimated recurrent mutation events at the locus.

Epidemiological data from Madera County indicated that the G29140U variant is replication competent, retains its virulence, and adequately transmits within and between different communities (see Text S1 in the supplemental material).

Our data show that even in areas of high SARS-CoV-2 community spread, replication-competent mutations that impair RT-PCR performance can emerge and spread, leading to reduced test sensitivity and potentially underdiagnosis if only one viral target is used. Since mutations have been described in primer/probe-binding regions of all published SARS-CoV-2 diagnostic assays (6), our findings strongly support continuous monitoring for mismatches and the routine use of at least two targets for SARS-CoV-2 detection by RT-PCR to avoid false-negative results.

### Data availability.

All code and *C_T_* data are available in our Github repository: https://github.com/czbiohub/polymorphism_sarscov2_diagnostics. Sequence data are available via GISAID; see also Table S1 for a list of sequences used.

## Supplementary Material

Supplemental file 1

Supplemental file 2
