# Supplementary material for: Identification of a Polymorphism in the N Gene of SARS-CoV-2 That Adversely Impacts Detection by Reverse Transcription-PCR
Source: J Clin Microbiol. 2020 Dec 17;59(1):e02369-20. doi: 10.1128/JCM.02369-20 (PMC7771441; doi:10.1128/JCM.02369-20)
Supplement: Supplemental file 1 [file JCM.02369-20-s0001.pdf]

Supplementary Text

Identification of a polymorphism in the N gene of SARS-CoV-2 that adversely impacts detection by RT-PCR.

Manu Vanaerschot[1], Sabrina A. Mann[1,2], James T. Webber[1], Jack Kamm[1], Sidney M. Bell[3], John Bell[4], Si Noon Hong[5], Minh Phuong Nguyen[5], Lienna Y. Chan[1], Karan D. Bhatt[1], Michelle Tan[1], Angela M. Detweiler[1], Alex Espinosa[4], Wesley Wu[1], Joshua Batson[1], David Dynerman[1], CLIAHUB Consortium, Debra A. Wadford[4], Andreas S. Puschnik[1], Norma Neff[1], Vida Ahyong[1], Steve Miller[2], Patrick Ayscue[1], Cristina M. Tato[1], Simon Paul[5], Amy Kistler[1], Joseph L. DeRisi[1,2], Emily D. Crawford[1,2,#]

[1]Chan Zuckerberg Biohub, San Francisco, California, USA; [2]University of California, San Francisco, California, USA; [3]Chan Zuckerberg Initiative, Redwood City, California, USA (Sidney M. Bell); [4]California Department of Public Health, Richmond, California, USA; [5]Department of Public Health, Madera, California, USA; [#]emily.crawford@czbiohub.org.

Supplementary Methods

SARS-CoV-2 detection by qPCR. The RNA of nasopharyngeal or oropharyngeal swabs, collected in DNA/RNA Shield medium (Zymo Research, USA), was extracted using the Quick-DNA/RNA Viral MagBead kit (Zymo Research, USA) and eluted in 40 µl of nuclease-free water. The RT-PCR assays included of 3 single reactions to detect the viral N gene (NIID_2019-nCov_N_F2, NIID_2019-nCov_N_R2ver3, and NIID_2019-nCov_N_P2 as described by (1)), the viral E gene (E_Sarbeco_F, E_Sarbeco_ R, and E_Sarbeco_ P1 as described by (2)) and the human control RNAse P gene (as described by (3)). All assays were performed in hard-shell thin-wall 384-well PCR plates (Bio-rad, USA) with a total volume of 10 µl per reaction, including 4 µl of eluted RNA and using the Luna Universal Probe One-Step RT-qPCR kit (NEB, USA). The PCR cycling protocol included 45 cycles and an annealing and elongation step at 58°C. A sample was considered positive for SARS-CoV-2 if one or both of the viral targets were detected with a C_t value <40, and the RNAseP Ct < 38.

Sanger sequencing of N gene fragment. The 28,928-29,521 nt genomic region of SARS-CoV-2 (NC_045512), containing the N gene fragment detected in our RT-PCR assay, was PCR-amplified using 5’-CTTGCTTTGCTGCTGCTTGA-3’ as the forward primer and 5’-TGAGTCAGCACTGCTCATGG-3’ as the reverse primer for 30 cycles using an annealing temperature of 67°C. Amplicons were purified using AMPure XP beads (Beckman Coulter, USA) at a ratio of 1.5, and sent to Genewiz (USA) for Sanger sequencing.

Whole genome sequencing and phylogenetic analysis of SARS-CoV-2 samples. We randomly selected 20 samples with increased ΔCt(N-E) (suspected mutants) and 11 samples with a minimal ΔCt(N-E) (suspected wild-types) from a subset of Madera County samples that showed E gene Ct

values <30. SARS-CoV-2 genome sequences from Madera County samples were recovered via Primal-Seq Nextera XT version 2.0 (modified from (4)), with the ARTIC Network v3 primers (5)), followed by paired-end 150bp sequencing on the Illumina platform. Reads were aligned to the reference genome (genbank accession MN908947.3) with minimap2 (6), samtools (7) to generate a pileup, and ivar (8) to trim primers. A phylogenetic tree was estimated with Nextstrain's ncov repository (9), using MAFFT (10), IQ-tree (11) and treetime (12); specific parameter settings are documented in the configuration files available on Github. Additional samples from GISAID were selected by searching CoV-GLUE for sequences with the Q289H mutation, and by using Nextstrain's sparse subsampling scheme to select additional wildtype samples for context. The figure of the phylogenetic tree was generated with the R package ggtree (13). Sequence data downloaded from GISAID is available in Supplementary Table 3, all code and sequences used for analyses and figure generation is publicly available (14).

Supplementary Results

Madera County is currently experiencing significant community spread of COVID-19, with a 14-day incidence rate of 378 cases per 100,000 population during July 1 — July 14, when the first mutant sample was observed (15). Out of 202 total samples from this county, 45 (22.3%) carried the G29140U mutation. These originated from four of thirteen mobile community testing sites in Madera County that covered both residential and also rural areas where primarily agricultural workers reside, suggesting broad geographic distribution of cases within the county. Nine of the samples originated from a single outbreak at a skilled nursing facility. Limited case investigation data from Madera County patients indicate that 17 of 19 individuals reporting symptom status did

exhibit COVID-19 symptoms (89%) with two deaths (4.8%). Cases occurred in individuals in a mix of professions [out of 14 reporting: 1 healthcare worker (7%); 1 laborer (7%); 2 craftsman (14%); 2 retired (14%); 3 unemployed (21%); 4 farmworkers (29%)] and predominantly among Hispanic or Latinx persons [13/18 (72%)]. These data support that the G29140U variant is replication-competent in this specific genetic background and adequately transmits within and between different communities.

Supplementary references

1. Shirato K, Nao N, Katano H, Takayama I, Saito S, Kato F, Katoh H, Sakata M, Nakatsu Y, Mori Y, Kageyama T, Matsuyama S, Takeda M. 2020. Development of genetic diagnostic methods for detection for novel coronavirus 2019(nCoV-2019) in Japan. Jpn J Infect Dis 73:304–307.
2. Corman VM, Landt O, Kaiser M, Molenkamp R, Meijer A, Chu DKW, Bleicker T, Brünink S, Schneider J, Schmidt ML, Mulders DGJC, Haagmans BL, Van Der Veer B, Van Den Brink S, Wijsman L, Goderski G, Romette JL, Ellis J, Zambon M, Peiris M, Goossens H, Reusken C, Koopmans MPG, Drosten C. 2020. Detection of 2019 novel coronavirus (2019-nCoV) by real-time RT-PCR. Eurosurveillance 25:2000045.
3. Lu X, Wang L, Sakthivel SK, Whitaker B, Murray J, Kamili S, Lynch B, Malapati L, Burke SA, Harcourt J, Tamin A, Thornburg NJ, Villanueva JM, Lindstrom S. 2020. US CDC Real-Time Reverse Transcription PCR Panel for Detection of Severe Acute Respiratory Syndrome Coronavirus 2. Emerg Infect Dis 26:1654–1665.
4. Quick J, Grubaugh ND, Pullan ST, Claro IM, Smith AD, Gangavarapu K, Oliveira G, Robles-Sikisaka R, Rogers TF, Beutler NA, Burton DR, Lewis-Ximenez LL, De Jesus JG,

Giovanetti M, Hill SC, Black A, Bedford T, Carroll MW, Nunes M, Alcantara LC, Sabino EC, Baylis SA, Faria NR, Loose M, Simpson JT, Pybus OG, Andersen KG, Loman NJ. 2017. Multiplex PCR method for MinION and Illumina sequencing of Zika and other virus genomes directly from clinical samples. Nat Protoc 12:1261–1276.

5. Artic Network. https://artic.network/resources/ncov/ncov-amplicon-v3.pdf.

6. Li H. 2018. Minimap2: Pairwise alignment for nucleotide sequences. Bioinformatics 34:3094–3100.

7. Li H, Handsaker B, Wysoker A, Fennell T, Ruan J, Homer N, Marth G, Abecasis G, Durbin R. 2009. The Sequence Alignment/Map format and SAMtools. Bioinformatics 25:2078–2079.

8. Grubaugh ND, Gangavarapu K, Quick J, Matteson NL, De Jesus JG, Main BJ, Tan AL, Paul LM, Brackney DE, Grewal S, Gurfield N, Van Rompay KKA, Isern S, Michael SF, Coffey LL, Loman NJ, Andersen KG. 2019. An amplicon-based sequencing framework for accurately measuring intrahost virus diversity using PrimalSeq and iVar. Genome Biol 20:8.

9. Hadfield J, Megill C, Bell SM, Huddleston J, Potter B, Callender C, Sagulenko P, Bedford T, Neher RA. 2018. NextStrain: Real-time tracking of pathogen evolution. Bioinformatics 34:4121–4123.

10. Katoh K, Standley DM. 2013. MAFFT multiple sequence alignment software version 7: Improvements in performance and usability. Mol Biol Evol 30:772–780.

11. Nguyen LT, Schmidt HA, Von Haeseler A, Minh BQ. 2015. IQ-TREE: A fast and effective stochastic algorithm for estimating maximum-likelihood phylogenies. Mol Biol Evol 32:268–274.

12. https://github.com/neherlab/treetime.

13. Yu G. 2020. Using ggtree to Visualize Data on Tree-Like Structures. Curr Protoc Bioinforma 69:e96.

14. https://github.com/czbiohub/polymorphism_sarscov2_diagnostics.

15. Madera County Department of Public Health. 2020. https://maderacounty.maps.arcgis.com/apps/opsdashboard/index.html#/5c3f6e5ce44d4160b46233ae1fa1e919.
